# Neurodevelopmental disorders in children: the role of MRI in early detection and intervention planning

**DOI:** 10.3389/fnins.2026.1758568

**Published:** 2026-03-11

**Authors:** Chen Hua, Xue-Ling Wang, Hui Sheng

**Affiliations:** 1 Department of Child Mental Health, Yantaishan Hospital, Yantai, China; 2 Department of Radiology, Yantaishan Hospital, Yantai, China

**Keywords:** brain imaging, early detection, machine learning, neurodevelopmental disorders, pediatric MRI

## Abstract

A group of diseases caused by disruptions in early brain maturation is collectively known as neurodevelopmental disorders (NDDs). These are characterized by persistent deficits in cognition, behavior, social or motor functioning. The heightened neuroplasticity could be modulated by appropriate intervention during early childhood. Therefore, early detection of NDDs is critical to improve long term developmental outcomes. However, conventional and behavioral studies are insufficient to detect the subtle early alterations, causing diagnostic delays. So, for NDDs, magnetic resonance imaging (MRI) serves as a critical tool for elucidating neurochemical, microstructural, and functional abnormalities. It has the potential to detect the alterations associated with different NDDs including autism spectrum disorder, attention deficit/hyperactivity disorder, genetic/metabolic syndromes, cerebral palsy, and developmental delay. Multiple modalities of MRI such as diffusion imaging, quantitative MRI, resting state functional MRI, and spectroscopy are applied for these disorders. Collectively, these MRI modalities, machine learning and integrative genomic approaches offer promising biomarkers for early detection and risk stratification of NDDs. This review highlights the current evidence on the bases of pediatric MRI approaches, early predictive biomarkers, disease specific findings, and translational applications.

## 1 Introduction

Neurodevelopmental disorders (NDDs) refer to a class of disorders which arise due to abnormal brain development in early life. These disorders are normally present in childhood and characterized by persistent deficits in behavior, social functioning, cognition, and motor skills. Cerebral palsy (CP), intellectual disability, attention deficit/hyperactivity disorder (ADHD), autism spectrum disorder (ASD), and developmental coordination disorder are considered as major NDDs. These disorders usually co-occur, such as ADHD and ASD often exist together, and complicate clinical trajectories (Hollingdale et al., 2024; Ogundele and Morton, 2025). The magnetic resonance imaging (MRI) studies reveals that, although certain neural variations overlap across diagnoses but distinct brain structure and functional connectivity differences are present in each condition (Kangarani-Farahani et al., 2022; Wan et al., 2024). The NDDs are inherently multifactorial such as environmental exposures, genetic variation, and perinatal complications all contribute to risk. This etiological heterogeneity is revealed in brain level variability, where neuroimaging studies indicate that even within a single diagnostic type, children can show distinct neural phenotypes (De Felice et al., 2015; Shan et al., 2022). The reported studies integrating the neuroimaging with transcriptomic and genetic data indicate that in children, ADHD and ASD are characterized by distinctive neuroanatomical signatures (Berg et al., 2023). As NDDs start in early life so these not only include behavioral syndromes but also fundamental disorders of brain development, with neurobiological changes. Advanced techniques that can non-invasively examine structure and function during development are required for understanding these developmental progressions (Gilmore et al., 2018; Reichard and Zimmer-Bensch, 2021). The NDDs such as ASD and ADHD manifest early in life but remain challenging to diagnose objectively due to heterogeneous clinical presentations and reliance on behavioral assessments alone. Reproducible, objective biomarkers that can be found before behavioral signs appear have the potential to revolutionize intervention planning and diagnostic periods. However, the clinical validity and generalizability requirements for regular practice have not yet been met by the potential biomarkers that have been found using neuroimaging, notably MRI (Wang et al., 2023; van der Meulen et al., 2024; Ramdial et al., 2025).

The early detection of NDDs is important because of the heightened neuroplasticity of the young brain. Intervention in the early stage of life can enhance gains because neural circuits are still being shaped and are more responsive to alteration. Specifically, the early behavioral interventions have been shown to improve long term outcomes in autism, language delay, and other neurodevelopmental conditions (Sullivan et al., 2014). Moreover, early detection also allows to monitor and manage medically comorbid conditions (such as epilepsy, sleep disorders, or metabolic abnormalities) before they exacerbate developmental disorders. However, in real practice, diagnosis is often delayed. Most of the children are only reliably diagnosed after age 4 or even later, at this time many critical periods of brain maturation have already passed (Zuckerman et al., 2017; Kentrou et al., 2019). Delays arise from several reasons such as limitations in caregiver, long waits for specialist assessment, and variability in symptom. Without reliable biomarkers, the clinical screening largely depends on behavior and it cannot fully manifest until later. In this context, the main biomarkers that reflect underlying brain biology rather than only behavior can decrease the diagnostic process (Goldani et al., 2014; Wang B. M. et al., 2025). MRI is not routinely used as a first-line diagnostic or screening technique prior to neuropsychological and behavioral examinations, despite the fact that it has shown significant potential in detecting early neurobiological changes linked to NDDs. Rather than a lack of scientific significance, this is a reflection of practical clinical, methodological, ethical, and financial limitations. First, MRI results are still mostly probabilistic and group-level rather than defined for individual diagnostic levels in early childhood, while clinical diagnostic frameworks are symptom-based and internationally recognized instruments are affordable, validated, and frequently used (Lord et al., 2020). Second, the viability and risk-benefit analysis of MRI in asymptomatic or moderately symptomatic people are constrained by pragmatic and ethical considerations (Copeland et al., 2021). Finally, rather than being a universal early screening tool, MRI is frequently saved for kids with additional neurological symptoms or an ambiguous clinical picture due to financial and accessibility limitations (Barkovich et al., 2019).

The traditional diagnosis of NDDs mainly relies on structured interviews, behavioral assessments, and standardized rating scales. For autism, the instruments such as the Autism Diagnostic Observation Schedule (ADOS) and Autism Diagnostic Interview Revised (ADI-R) are considered gold standards (Thurm et al., 2016; Lebersfeld et al., 2021). These tools mainly depend on observable behavior and caregiver reports, which may not be reliable in infants. Early indicators like modest motor delays and social isolation should not be considered as typical variation, particularly when there are no apparent symptoms present. Moreover, the behavioral assessments can be confounded by external factors such as cultural differences, socioeconomic status, language background, and even caregiver perceptions can bias reporting (Huda et al., 2024). There is a scarcity of trained developmental pediatricians, child neurologists, and clinical psychologists in many developing countries, which limits access to timely evaluations. These limitations highlight a persistent need for complementary instruments that provide biologically based insights beyond behavior (Roy et al., 2023; Muthiga et al., 2025).

MRI has emerged as a powerful and non-invasive window into the developing brain. Rather than focusing only on the anatomy, modern MRI techniques can interrogate microstructure, connectivity, metabolism, and perfusion domains which are highly relevant to NDDs (Dubois et al., 2021; Tang et al., 2022). Structural MRI has revealed different trajectories of brain growth for ASD and ADHD. A study of cortical thickness found both common and disorder specific patterns in children with ASD and ADHD. In showed cortical thinning in some regions and thickening in others suggest differential maturational delays or divergence (You et al., 2024). Diffusion MRI, particularly diffusion tensor imaging (DTI), has been highly informative. The DTI meta-analyses across NDDs show consistent alterations in white matter microstructure. The decreased fractional anisotropy (FA) in the corpus callosum in case of ASD and ADHD, as well as increased mean diffusivity (MD) in posterior thalamic radiations in ASD suggested disrupted maturation of long-range areas (Zhao et al., 2022). In addition to DTI, infants are treated using advanced diffusion techniques such as constrained spherical deconvolution (CSD), diffusion kurtosis imaging (DKI), and neurite orientation dispersion and density imaging (NODDI) (Kimpton et al., 2021; Zhao et al., 2021b). Compared to conventional DTI, these advance approaches provide particular biophysical models of microstructure and allow for the detection of subtle maturational dynamics with more specific way (DiPiero et al., 2023). The resting-state and task-based Functional MRI (fMRI) are useful to map the development of brain networks. In children with NDDs, the resting-state fMRI studies showed atypical connectivity patterns in networks which are involved in executive function, social cognition, and sensory processing. For example, in children with ADHD, resting-state fMRI demonstrated specific connectivity alterations correlated with behavioral measures of autism (Liu et al., 2023). Additionally, magnetic resonance spectroscopy (MRS) provides a complementary biochemical perspective by enabling the *in vivo* assessment of brain metabolites. In case of ASD, proton-MRS (H-MRS) studies reported typical levels of gamma aminobutyric Acid (GABA), glutamate (glu), and other important neurochemicals (Horder et al., 2018; Kubota et al., 2025). Although there is potential for multimodal MRI to reveal the neurological underpinnings of NDDs and to guide risk assessment and tailored treatment, there are numerous challenges to implementation. Replication, individual outcome prediction accuracy, and consistency between scanners and cohorts are frequently lacking in reported biomarker signals. Furthermore, few studies show prospective predictive potential or enhance decision-making beyond current clinical evaluations, raising doubts about the clinical value of early MRI results (Wang et al., 2023; van der Meulen et al., 2024).

Despite a number of narrative and systematic reviews detailing structural and functional MRI changes in NDDs, critical evaluations concentrating on clinical readiness and translational repeatability are still lacking. The reliability of certain MRI biomarkers across independent cohorts is seldom evaluated by previous studies, which usually indicate relationships but do not rigorously analyse where the evidence is inconsistent, weak, or limited by methodological heterogeneity (Wang et al., 2023; van der Meulen et al., 2024). This review specifically integrates evidence from multimodal MRI studies, highlights methodological limitations such as small sample sizes or inconsistent acquisition protocols, and identifies barriers to clinical adoption including low predictive validity, lack of external validation, and limited applicability outside research settings.

## 2 Neurodevelopmental disorders in children

### 2.1 Autism spectrum disorder (ASD)

**Figure 1:**
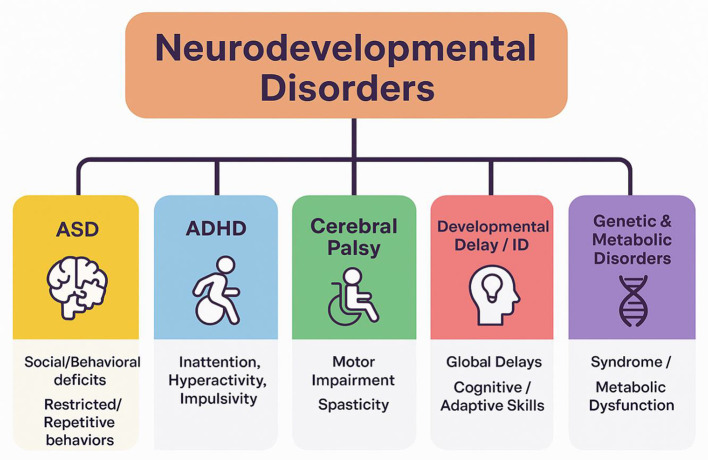
The classification of the main neurodevelopmental disorders with their primary domains of impairment.

While structural MRI is widely used in research to explore neurobiological alterations in ASD, evidence supporting its routine clinical use remains limited. Current practice guidelines do not recommend routine MRI in ASD unless specific clinical indications exist, contrasting with a growing research focus on multimodal imaging biomarkers (Byrne et al., 2023). ASD is a typical NDDs characterized by persistent deficits in social communication and interaction, as well as restricted, repetitive behaviors in early childhood. ASD exhibits substantial phenotypic and neurobiological heterogeneity, underpinned by complex genetic, epigenetic, and environmental factors (Hodges et al., 2020; Leisman et al., 2025). Neuroimaging studies, especially with MRI, have revealed a variety of brain alterations in ASD but the clinical yield of routine MRI remains modest. An ASD study of 181 children reported only 7.2% clinically significant neuroimaging abnormalities. These were much more likely when there was a neurological examination abnormality or a known genetic/metabolic condition (Byrne et al., 2023). H-MRS studies further show the elevated glutamate peaks, particularly in the cerebellum. It has been observed in ASD patients which suggesting that excitatory inhibitory imbalance may play a role (Ajram et al., 2019; Johnson et al., 2023). Nevertheless, studies comparing ASD with other neurodevelopmental disorders suggest that although there may be some overlap in brain features, each disorder often displays distinct neural correlates. As a review found that the majority of imaging abnormalities (about 77%) were disorder specific rather than shared, which highlights the importance of imaging biomarkers for differential pathways (Kangarani-Farahani et al., 2022). Usually, the routine MRIs in ASD yield incidental findings, such as benign enlargement of the subarachnoid spaces, persistent cavum septum pellucidum, or mega cisterna magna. These findings correlate with symptom severity but are not disease specific (Afify et al., 2025). Classification of major NDDs highlighting their primary domains, is shown in Figure 1.

Recent clinical studies show that routine MRI findings in ASD are uncommon and often incidental, with abnormalities identified in almost 7% of cases and predominantly in children with abnormal neurological signs or genetic/metabolic conditions, rather than ASD symptoms. Therefore, consensus statements from pediatric neurology societies advise that routine MRI is not indicated in typical ASD presentations (Byrne et al., 2023).

Structural MRI findings across cohorts have identified heterogeneity in cortical and subcortical morphology in ASD, including differences in whole brain volume, cortical thickness, and corpus callosum size compared to controls. However, results vary and show inconsistent effects across studies (Pagnozzi et al., 2018; Xie et al., 2023). Although generalizability and clinical applicability are yet unknown, systematic research employing resting-state fMRI shows little potential for applying machine learning techniques to distinguish ASD vs. usual development (Santana et al., 2022). Other studies highlight that combining structural, functional, and metabolic MRI can improve characterization of neural network disruptions in ASD, but such biomarkers are not yet validated for clinical diagnosis (Wang et al., 2023). Although promising machine learning and multimodal integration techniques across modalities indicate promise for research-based neurobiological biomarkers in ASD, their clinical translational relevance is still constrained by a lack of standardization, small sample numbers, and study design inconsistency (Traut et al., 2022). Thus, most imaging findings reflect neural circuitry alterations that cut across diagnostic categories, supporting dimensional frameworks such as the Research Domain Criteria (RDoC) rather than categorical, ASD-specific biomarkers. The detail of common NDDs in children is also given in Table 1.

**Table 1:** Overview of the major neurodevelopmental disorders along with epidemiology and clinical features.

**Disorder**	**Occurrence**	**Clinical features**	**Typical age**
ASD	1%−2% Globally	Social communication deficits, restricted behaviors, sensory sensitivities	Early childhood (2–3 years)
ADHD	5%−7% of children	Inattention, hyperactivity, impulsivity	Before age 12
CP	2–3 per 1,000 live births	Motor impairment, spasticity, gait abnormalities, possible cognitive impairment	Birth or early infancy
Developmental delay/ intellectual disability	1%−3%	Global delays in motor, language, cognitive skills; impaired adaptive functioning	Early childhood
Genetic and metabolic disorders	Rare; varies by condition	Specific neurodevelopmental phenotypes (e.g., Down syndrome, phenylketonuria)	Variable, often early

### 2.2 Attention deficit/hyperactivity disorder (ADHD)

ADHD is one of the most common pediatric neurodevelopmental disorders, which is characterized by hyperactivity, inattention, and impulsivity. Neurobiologically, diffusion MRI (particularly DTI) studies implicate disrupted white matter microstructure as a core feature. A reported study of 46 diffusion MRI studies in children and adolescents with ADHD found consistent alterations in tracts such as fronto-striatal pathways, the corpus callosum, cingulum bundle, superior longitudinal fasciculus, internal capsule, thalamic radiations, and corona radiate. These analyses further showed global underconnectivity across functionally specialized networks (Connaughton et al., 2022). In younger children (4 to 7 years), structural and diffusion imaging can predict ADHD diagnosis and symptoms. A predictive modeling study showed that diffusion measures in the inferior frontal gyrus (Öztekin et al., 2022). The fMRI adds further insight as the dynamic functional connectivity studies using resting-state fMRI demonstrate that children with ADHD show greater temporal variability in connectivity across networks. Especially in fronto-temporal, cingulo-parietal, and fronto-parietal regions, possibly linking to attentional fluctuations and executive control deficiency (Misra and Gandhi, 2023). Quantitative myelin imaging is also emerging, as a recent conference abstract compared synthetic MRI and DTI measures of myelin in children with ADHD. It suggested that synthetic MRI may better quantify myelin volume fraction, which could refine our understanding of myelination deficits in ADHD (Lin et al., 2024). A recent study also highlighted that MRI combined with machine learning is gaining traction for ADHD classification, though challenges remain due to heterogeneity, sample sizes, and model generalizability (Ashraf et al., 2024). Multimodal imaging in ADHD shows a coherent profile of atypical cortical development, persistent white matter microstructural disruptions (especially in fronto-striatal and commissural tracts), and altered intrinsic functional connectivity within and between canonical networks (i.e., DMN, salience, executive networks). These convergent findings suggest consistent neurodevelopmental differences that can inform biomarker discovery (Wang et al., 2026a).

### 2.3 Cerebral palsy (CP)

The CP represents a group of permanent, non-progressive motor disorders resulting from early brain injury. It is commonly associated with additional neurodevelopmental comorbidities such as ASD and ADHD. In CP, the conventional MRI reveals abnormalities in up to 83 % of cases, most frequently white matter injury, gray matter damage, or malformations (Korzeniewski et al., 2008). A population-based MRI neuropsychiatric study found that among children with CP, comorbid autism (30%) and ADHD (31%) are common across different lesion types, but prevalence varies by injury timing and location. Particularly, the children with predominant white matter injury showed higher rates of ASD, while vascular infarct patterns were associated with higher ADHD prevalence (PÅhlman et al., 2022). Furthermore, combining MRI phenotypes with genetic analysis can guide etiological work-up. For example, a study argues that MRI classification systems in CP help to determine when genetic testing is necessary. The malformations or normal MRI findings suggest a stronger genetic etiology, while acquired patterns (white and gray matter injury) may reflect perinatal insults (Horber et al., 2021). A study from a large CP registry reported that not all children with CP show MRI abnormalities. Therefore, CP and a completely normal MRI, indicating the need for advanced imaging techniques and perhaps further genetic/metabolic evaluation (Springer et al., 2019). In CP, MRI frequently demonstrates PWMI and other WM injury patterns, which strongly correlate with motor and cognitive outcomes, reflecting disruption of typical myelination processes (Yuan et al., 2025).

### 2.4 Developmental delay (DD) and intellectual disability (ID)

Global developmental delay (GDD) and intellectual disability (ID) are broad categories encompassing children with delayed milestones, lower cognitive functioning, and a deficiency in adaptive behaviors. The role of MRI in this group has been studied extensively. It showed that MRI in children with GDD or isolated intellectual disability reported abnormal neuroimaging findings in about 38% of cases. However, the diagnostic yield (i.e., finding a clear etiological lesion) was lower (7.9%) across the studied cohorts (Murias et al., 2017). MRI has identified morphologic abnormalities in children with ASD along with low functioning autism and in non-syndromic intellectual disability, including findings like mega cisterna magna and hypoplastic corpus callosum (Erbetta et al., 2015). In a cohort of 471 children with mild ID, only 12.5% had significant MRI abnormalities, and almost all those cases had additional neurological signs (seizures, movement disorder, dysmorphic features, etc.). Only 1% of children had abnormal MRI without any other indication, suggesting routine MRI in mild ID without other clinical red paper chain may not be justified (Jussila et al., 2021). From a care perspective, mental health in children with neurogenetic disorders associated with ID is notable. A meta-analysis of psychiatric comorbidity found significantly elevated prevalence in genetic syndromes such as Down, Fragile X, 22q11.2 deletion, and Prader Willi, highlighting that imaging must often be integrated with genetic, behavioral, and psychiatric evaluation (Glasson et al., 2020). In pediatric genetic disorders associated with ID, MRI often shows symmetrical WM signal abnormalities, corpus callosum thinning, and delayed or hypomyelination, indicating core disruptions in structural connectivity (Oikarainen et al., 2025).

### 2.5 Genetic and metabolic disorders affecting neurodevelopment

A subset of neurodevelopmental disorders originates from inborn errors of metabolism (IEM) and other genetic syndromes, which can manifest as global delays, intellectual disability, seizures, or other neurological features. Neuroimaging plays a critical in this context, as MRI and MRS usually reveal characteristic patterns in numerous IEMs, that can facilitate early diagnostic suspicion before genetic confirmation (Lai et al., 2022). For instance, MRI in metabolic disorders may reveal characteristic alterations including basal ganglia hyperintensities, diffusion abnormalities, or evolving white-matter injury. Correspondingly, MRS in these patients often shows metabolite peaks showed the elevated glutamine/glutamate, lactate, reduced N-acetyl aspartate providing valuable biochemical signatures (Lai et al., 2022; Eda et al., 2025). Moreover, in inherited metabolic epilepsies (IMEs), neuroimaging serves not only diagnostic but also prognostic and monitoring roles: a recent review highlights that some IMEs show distinct MRI or MRS patterns, and advanced modalities (PET, DTI, g-ratio mapping) further enrich understanding of disease pathophysiology and response to therapy (Tokatly Latzer et al., 2025). From a translational perspective, case-based neuroimaging-MRS correlations in treatable neurometabolic disorders (e.g., biotin-thiamine–responsive basal ganglia disease, creatine deficiency syndromes) have directly informed therapeutic decisions, underscoring the importance of imaging in early diagnosis and management (Eda et al., 2025). The emerging work using multimodal MRI coupled with machine learning classification shows promise for early detection and subtyping of NDD features, though generalizability remains limited by sample heterogeneity and algorithm complexity (Zhang-James et al., 2023; Ouyang et al., 2024). MRI biomarkers for pediatric brain development are given in Table 2.

**Table 2:** Selected MRI biomarkers of atypical brain development.

**Biomarker**	**Disease/Target**	**Detection system (modality and metric)**	**References**
Early cortical folding and volumetry	General early brain maturation	Structural MRI (T_1_ volumetry, cortical morphometry)	Ouyang et al., 2019
Neurite density (NODDI)	Infancy normative mapping	Diffusion MRI (NODDI)	Zhao et al., 2021a
Regional myelin density (early)	Language outcomes prediction	Quantitative myelin MRI	Corrigan et al., 2022
Intracortical myelination patterns	ASD risk/atypical development	Myelin-sensitive structural MRI	Chen et al., 2022
Corpus callosum & long-range tract FA/MD	ASD, ADHD	Diffusion MRI (DTI metrics: FA and MD)	Li et al., 2017
Resting-state network maturation (fronto-temporal)	Language/social cognition disorders	fMRI (network topology, connectivity strength)	Chen et al., 2021
Whole-brain normative divergence scores	Transdiagnostic risk stratification	Multimodal MRI and normative modeling	Jin and Wang, 2024
Metabolite profiles (Glu/GABA, lactate)	ASD & metabolic disorders	H-MRS metabolite peaks	Li et al., 2017
Myelination gradients (global vs regional)	General developmental prediction	Quantitative MRI, longitudinal studies	Grotheer et al., 2022
Advanced diffusion (NODDI) in infants	Improved microstructural specificity	NODDI (neurite density, orientation dispersion)	Niu et al., 2025
Aggregated MRI diagnostic performance	ASD diagnostic biomarker meta-analysis	Multimodal MRI and ML meta-analysis	Schielen et al., 2024
Cortical thickness, volume	ASD	T1 morphometry (cortical thickness)	Lucibello et al., 2022
FA, MD in long tracts	ADHD	TBSS, tractography	Parlatini et al., 2023
Network connectivity	ASD, language disorders	rs-fMRI (graph metrics)	Wang et al., 2023
Glu/GABA, NAA	ASD	^1^H-MRS quantification	Thomson et al., 2024
NODDI neurite density	Infant microstructure	NODDI modeling	Weber et al., 2022
Cortical complexity	General NDD detection	T1 cortical metrics	Abujamea et al., 2023
Regional perfusion	Language, attention deficits	ASL CBF quantification	Wang et al., 2023
Myelin indices (R_1_, MWF)	Cognitive outcome prediction	qMRI mapping	Marano et al., 2025
Tract-specific microstructure	ASD	Along-tract FA/MD mapping	Kim et al., 2025
Aggregated biomarkers	ASD classification	Structural, DTI, fMRI, ML	Schielen et al., 2024
Lactate, NAA	Metabolic disorders	H-MRS diagnostic peaks	Thomson et al., 2024
Dynamic connectivity metrics	ADHD	Time-varying connectivity	Weber et al., 2022
FA reduction summary	ASD	Meta-analytic TBSS	Zhao et al., 2022
Volume deficits in CHD	Developmental risk	Neonatal, infant T_1_ volumetry	Fu et al., 2025
Hemodynamic connectivity	Infant ASD screening	fNIRS resting-state	Bi et al., 2025

## 3 MRI modalities used in evaluating neurodevelopmental disorders

MRI offers a range of modalities that together map macroscopic anatomy, microstructure, functional dynamics, and brain chemistry each providing complementary biomarkers for NDDs. However, pediatric specific methodological considerations are emphasized because the developmental stage critically shapes signal interpretation (Lewis et al., 2025).

### 3.1 Structural, diffusion, and functional MRI

High resolution T_1_-weighted structural MRI considered as powerful tool for *in vivo* morphometry of cortical thickness, gray/white matter volumes. The approaches such as total and regional brain volumes, cortical thickness, surface area, and gyrification index capture normative maturational patterns (rapid volumetric growth in the first two postnatal years, followed by regionally variable pruning and cortical thinning) and deviations associated with disorders (Knickmeyer et al., 2008; Gilmore et al., 2018). Cortical thickness have been linked to cognitive and behavioral phenotypes (for example, atypical early cortical thickening or delayed thinning in ASD and some genetic syndromes), and volumetric asymmetries in basal ganglia or hippocampus have been reported across ADHD, intellectual disability and metabolic conditions. Prominently, age and scanner harmonized atlases are necessary to distinguish true pathology from normal developmental variance (Lucibello et al., 2022). White matter microstructure and connectivity were studied by diffusion MRI, historically dominated by diffusion tensor imaging (DTI) metrics, probes white matter microstructure and tract coherence (Nucifora et al., 2007; Tamnes et al., 2018). DTI meta-analyses in pediatric populations reveal consistent alterations in commissural, projection and association tracts across ADHD and ASD, implicating pathways supporting attention, language and interhemispheric integration. Recently, advanced diffusion models (NODDI, DKI, CSD) provide greater specificity and also improve sensitivity to early maturational processes in infants and toddlers specially for early detection paradigms. Along-tract mapping and connectomic metrics (edge density, network efficiency) further translate microstructural changes into network level interpretations relevant for behavioral phenotypes (Weber et al., 2022; Parlatini et al., 2023). The fMRI interrogates task induced activation and intrinsic spontaneous activity. Resting-state fMRI approaches have been particularly useful in infants and young children because they avoid task compliance requirements. Resting-state fMRI studies show maturation from local, short-range connectivity toward distributed, long-range networks (default mode, salience, fronto-parietal) and have identified atypical patterns associated with ASD, ADHD, and language disorders (Uddin et al., 2013; Gao et al., 2017). Task-based fMRI act as a valuable tool for mapping domain specific activations (language, motor planning, and inhibitory control) and for tracking intervention related plasticity. Methodological precision in motion correction, age appropriate preprocessing, and consideration of sleep and sedation states is essential in pediatric fMRI to avoid spurious connectivity findings (Nagai et al., 2024).

### 3.2 Magnetic resonance spectroscopy (MRS) and advanced and emerging MRI techniques

The H-MRS non-invasively measures regional metabolite concentrations (N-acetylaspartate, choline, creatine, glutamate/glutamine, GABA, lactate) that index neuronal integrity, membrane turnover, and excitatory inhibitory balance (Narayana et al., 2019). Meta-analytic evidence in children with ASD indicates reduced GABA and N-acetylaspartate (NAA) in selected regions and altered Glu/GABA ratios consistent with hypothesized excitatory inhibitory dysregulation. In metabolic and mitochondrial disorders, MRS can provide pathognomonic chemical signatures (e.g., lactate peaks), guiding rapid diagnostic and therapeutic decisions. The quantitative MRS protocols and standardized voxel placements improve interstudy comparability and clinical utility (Thomson et al., 2024). The advanced and emerging MRI techniques (ASL, qMRI, fNIRS integration) expand the biomarker palette (Yang and Wang, 2025; Zhang et al., 2025, 2026). Arterial spin labeling (ASL) measures cerebral blood flow non-invasively and has been applied to detect perfusion anomalies linked to language and attention deficits. The qMRI parameters, including the longitudinal relaxation rate, magnetization transfer ratio (MTR), myelin water fraction, and related putative myelin indices demonstrate significant correlations with neurodevelopmental milestones and cognitive performance outcomes (Yerys et al., 2018; Schmidbauer et al., 2024). Hybrid and cross-modal integrations [multimodal MRI, simultaneous EEG-fMRI, or MRI along with functional near-infrared spectroscopy (fNIRS)] improve robustness for infant studies where motion and scanner accessibility are limiting. ML frameworks that fuse multimodal features (structural, diffusion, functional, MRS, and qMRI) show promise for early classification and individualized risk stratification, but these require large, harmonized cohorts and external validation before clinical development (Wang et al., 2023). In short, each MRI modality contributes unique, age-sensitive biomarkers. The structural MRI quantifies macroscopic anatomy and cortical maturation, diffusion MRI reveals microstructural tract integrity, fMRI captures dynamic network function, MRS assesses neurochemistry, and ASL/qMRI/fNIRS augment sensitivity to perfusion and myelination. The translational challenge is integrating these measures into validated, developmentally normative frameworks that can reliably predict individual risk and treatment responsiveness (Marano et al., 2025).

Although multimodal MRI provides rich neurobiological information, its routine application in all children with suspected neurodevelopmental disorders is neither feasible nor clinically justified. Current evidence and expert consensus support a targeted, indication-driven approach, in which advanced MRI is reserved for specific clinical scenarios where imaging findings are likely to influence diagnosis, prognosis, or intervention planning (Krishna and McInnes, 2020; Copeland et al., 2021). Multimodal MRI is most clearly indicated when neurodevelopmental symptoms are accompanied by neurological red flags, including seizures, abnormal head circumference, focal neurological deficits, or developmental regression. In such cases, structural MRI, diffusion imaging, and magnetic resonance spectroscopy can identify malformations, white-matter injury, or metabolic abnormalities that are not detectable through behavioral assessment alone (Shevell et al., 2003; Murias et al., 2017).

## 4 MRI findings in major neurodevelopmental disorders

MRI has elucidated disorder specific and transdiagnostic brain signatures across major pediatric NDDs, yielding mechanistic insight and candidate biomarkers for early detection and prognosis. There are modality specific findings for ASD, ADHD, CP, developmental delay/intellectual disability (DD/ID), and genetic/metabolic disorders (Parellada et al., 2023). The results of MRI for different NDDs are provided in Table 3.

**Table 3:** MRI findings for different neurodevelopmental disorders.

**MRI mode**	**Biomarker**	**Disease**	**Detection system**	**Key findings**	**References**
Multimodal MRI review	Response biomarkers	ASD	MRI modalities synthesis	Catalogs candidate imaging biomarkers to guide interventions	Parellada et al., 2023
MRS review	Metabolite alterations	ASD and IEM	H-MRS	Reduced GABA/NAA in ASD, MRS diagnostic role in IEMs	Thomson et al., 2024
Prospective infant imaging	Early prediction	ASD	Structural and fMRI	Early brain differences in infants predict later ASD diagnosis	Dawson et al., 2023
Structural MRI review	Neuroanatomy	ASD	T_1_ morphometry	Lifespan cortical differences and developmental trajectories	Ecker et al., 2015
Systematic MRI in CP	Lesion classification	CP	Conventional MRI	High detection rate, lesion type predicts motor and cognitive outcome	Khan et al., 2022
DTI meta-analysis	FA reductions	ADHD	DTI	Consistent white matter microstructural reductions linked to ADHD symptoms	Parlatini et al., 2023
Neurochemistry meta	Glu/GABA	ASD	MRS meta-analysis	Evidence for excitatory/inhibitory imbalance in ASD	Horder et al., 2018
Systematic MRI yield	GDD/ID imaging	DD, ID	Conventional MRI	Pooled abnormality rates and clinical predictors of yield	Murias et al., 2017
Cohort preterm biomarkers	Early MRI predictors	Transdiagnostic NDD risk	Multimodal MRI	Early markers in preterm infants associated with later NDDs	Zhao et al., 2023
DTI infant study	Tract microstructure	At risk infants	Advanced diffusion	Infant white matter changes precede behavioral symptoms	Slaby et al., 2023
Imaging, genetics	MRI pattern yield	Genetic ID	MRI genetic testing	MRI patterns guide genetic diagnostic yield	Berry et al., 2025
Clinical evaluation GDD	MRI clinical utility	DD, ID	Clinical imaging audit	MRI abnormality correlates with additional neuro signs	Aldosari and Aldosari, 2024
White matter diffusivity	DD and ADHD comorbidity	DD, ADHD	TBSS	Shared and distinct white matter alterations in comorbid presentations	Slaby et al., 2023
Clinical MRI audit	Pediatric MRI in DD	DD clinical sample	Conventional MRI	Practical yields and indications for scanning	Magsi et al., 2025

### 4.1 MRI signatures in ASD, ADHD, and CP

ASD highlights atypical trajectories of brain growth, altered cortical organization, connectivity disturbances, and neurochemical imbalances. Group and longitudinal structural MRI studies report early brain overgrowth in a subset of children with ASD, region-specific cortical thickness deviations, and atypical cortical folding patterns that vary with age and cognitive level. The fMRI studies, particularly resting-state analyses, commonly detect altered connectivity within social cognitive networks, though effect directionality depends on age and analytic approach (Hazlett et al., 2011; Lee et al., 2025). H-MRS meta-analyses further indicate regional reductions in NAA and GABA alongside variable glutamate alterations, supporting the excitatory inhibitory imbalance hypothesis. Collectively, these multimodal signatures suggest that ASD involves early, regionally selective deviations in maturation that can predict later social and language outcomes (Dawson et al., 2023).

ADHD neuroimaging converges on delayed cortical maturation, volumetric reductions in fronto-striatal circuits, and pervasive white-matter microstructural alterations. Large diffusion MRI syntheses reveal consistent reductions in FA across projection, commissural and association fibers. Especially, alteration in the integrity of the corpus callosum, superior longitudinal fasciculus, and fronto-striatal tracts has been linked with deficits in attention and executive functioning. The resting-state fMRI studies report dysregulated fronto-parietal and cingulo-opercular network dynamics, while task fMRI highlights hypoactivation of inhibitory control regions during response inhibition tasks (Shaw et al., 2007; Parlatini et al., 2023). Recent multi-site diffusion meta-analyses demonstrate that white-matter measures correlate with symptom severity and cognitive metrics, reinforcing microstructure as a mechanistic substrate and potential target for early risk stratification (Parlatini et al., 2023).

The conventional MRI provides a high diagnostic yield, CP, and informs timing and etiology of the insult. Typical MRI patterns include periventricular white-matter injury in preterm infants, cortical, subcortical malformations, basal ganglia/thalami lesions after term hypoxic-ischaemic events, and focal infarcts producing hemiplegic CP. MRI lesion classification systems stratify risk for motor impairment and comorbidities such as epilepsy and cognitive impairment (Himmelmann et al., 2017, 2021). The lesion topography and extent also predict functional outcome and guide neurorehabilitation planning. Prominently, a subset of children with CP has normal conventional MRI, prompting the use of advanced diffusion, quantitative myelin imaging, and genetics to elucidate etiology (Khan et al., 2022).

### 4.2 MRI patterns in case of developmental delay and intellectual disability (GDD/ID) and in genetic and metabolic disorders

For children with GDD/ID, MRI yields variable diagnostic returns depending on severity and associated neurological signs. A study reported the abnormal MRI rates ranging widely (commonly 30% to 40%), with higher yields in moderate to severe. Common findings include cortical malformations, hypoxic-ischemic injury signatures, corpus callosum anomalies, and white-matter dysmyelination. While routine MRI in isolated mild ID has a lower yield, targeted imaging informed by phenotype and developmental trajectory remains valuable for etiologic clarification and for triaging genetic/metabolic testing (Table 3) (Murias et al., 2017). Through disorders MRI reveals both disorder-specific markers (e.g., lesion topography in CP; metabolic peaks in IEM) and transdiagnostic substrates (white-matter microstructure, network dysconnectivity) (MacKay et al., 2018; de Almeida Marcelino et al., 2025). The translational challenge is converting group level findings into robust, generalizable individual level biomarkers. This requires larger harmonized cohorts, longitudinal designs that anchor imaging to later functional outcomes, and rigorous external validation of predictive models. When combined with clinical, genetic, and behavioral data, MRI increasingly offers actionable information for early detection, prognosis, and intervention planning (Zhao et al., 2023). Many existing machine learning applications in pediatric neuroimaging are derived from relatively small, single-site datasets, which artificially elevate classification metrics and reduce external validity. Because of over fitting, decreased exposure to heterogeneity, and larger effect sizes, meta-analyses show that excellent performance reported in small cohorts tends to deteriorate as sample size grows. Furthermore, real generalizability issues may be concealed by inappropriate cross-validation without held-out data (Chen et al., 2023; Moreau et al., 2023).

## 5 MRI for early detection and risk stratification

One of the most compelling roles of MRI in pediatric NDDs is its potential for early detection and risk stratification, especially in infancy and toddlerhood. Brain imaging during this critical window can reveal biomarkers long before behavioral symptoms become overt, enabling proactive monitoring, targeted surveillance, and timely interventions (Hazlett et al., 2017). The longitudinal and prospective MRI studies in high risk infants have provided some of the strongest evidence that brain connectivity and morphometric signatures at 6 months can predict later diagnosis. In a resting-state functional connectivity MRI (fcMRI) at 6 months in infants at high familial risk for ASD, combined with an ML. This predicted autism diagnosis at 24 months with striking accuracy (positive predictive value; 100%, sensitivity; 82%, specificity; 100%) (Emerson et al., 2017). Another prospective cohort showed that extra axial cerebrospinal fluid (CSF) volume measured at 6 months, together with total brain volume, age, and sex, entered into a multivariate algorithm, predicted later ASD with moderate accuracy (sensitivity 66%, specificity 68%) (Wolff and Piven, 2020; Dawson et al., 2023). Structural growth trajectories in early infancy, such as accelerated surface area expansion between 6 to 12 months, followed by total brain volume increase from 12 to 24 months, have also emerged as predictive features in ML models (Wolff and Piven, 2020). The diffusion weighted imaging (DWI) also contributes key early risk markers. At 6 months, abnormalities in white-matter microstructure e.g., in the splenium of the corpus callosum and superior cerebellar peduncles are associated with later ASD diagnosis, with predictive values in single site infant (Wolff and Piven, 2020). Another study applied a multiscale white-matter connectome approach using hierarchical diffusion metrics (FA, mean diffusivity, fiber length) combined with support vector machines. It achieved 76% accuracy in classifying high risk infants at 6 months (Jin et al., 2015). Thus, even before behavioral criteria emerge, both connectivity and microstructure metrics carry prognostic information. The ML techniques have become central to realizing the predictive potential of MRI biomarkers. A recent study highlights that multimodal MRI collected in early childhood combined with ML substantially improves the power to forecast neurodevelopmental outcomes compared to single modality approaches (Ouyang et al., 2024). In ASD specifically, structural MRI has been widely studied from an ML perspective. A study of 3T structural MRI studies found that increased whole brain volume, especially in children under six, is a regular finding and may be leveraged by ML classifiers as a morphological biomarkers (Pagnozzi et al., 2018). More recently, deep learning using a contrastive variational autoencoder on MRI in young children (under 5 years) reported classification accuracies above 0.97, suggesting that latent features extracted by unsupervised deep models might identify subtle, disorder specific neuroanatomical signatures (Ma et al., 2024). These approaches are important from cross validation, large normative and high risk cohorts, and careful feature selection. Still, challenges remain in model generalizability, given developmental heterogeneity and scanner variability.

The integration of MRI with genetic and clinical data is recognized as a major tool to improve risk stratification. Neuroimaging genetics approaches combine imaging phenotypes with gene expression, polygenic risk scores, copy-number variants, or epigenetic data to identify biomarkers that lie closer to the molecular etiology of disorders (Nisar and Haris, 2023; Yu et al., 2025). A recent study showed how linking functional and structural brain variation to ASD risk genes via AI enables identification of early biomarkers, thereby bridging genotype, circuits, and behavior (Nisar and Haris, 2023). For example, a population cohort study recently demonstrated that polygenic risk scores for ADHD are associated with differences in brain volumes in children, suggesting a neurogenetic mechanism. In which the common genetic variation confers risk via brain structure (He et al., 2023). Another transdiagnostic family based project is explicitly integrating MRI with deep phenotyping of behavioral traits and genetic data across ASD and ADHD, aiming to identify neurobiologically distinct clusters that reflect shared and diverging risk pathways (Knott et al., 2021). These investigative approaches not only enhance predictive accuracy but also move toward more personalized risk models. These identify which infants may benefit from closer surveillance or early intervention based on their combined neuroimaging and genetic profile. Drawing on early biomarker discovery, predictive modeling, and multimodal integration, MRI based risk models are gradually emerging as translational tools. For ASD, a proposed level-2 screening paradigm envisions using MRI in infants already flagged as elevated risk. It combined with genetic and biomarker data to define an ultra-high risk group for early intervention (Wolff and Piven, 2020; Wang et al., 2023). Critically, the performance of these models depends on external validation, harmonized data collection, and clinical feasibility. A recent study discusses ethical and practical challenges, but underscores that multimodal MRI, ML, and genomics frameworks are rapidly approaching feasibility in research settings (Wang et al., 2023). Finally, the risk models must be embedded in longitudinal studies by combining imaging at multiple time points. ML models can not only predict diagnosis but also stratify trajectories, and informing personalized care pathways. Diagnostic accuracy of multimodal MRI biomarkers for early detection and outcome prediction in pediatric neurodevelopmental disorders is given in Table 4.

**Table 4:** Diagnostic accuracy of multimodal MRI biomarkers for early detection and outcome prediction in pediatric neurodevelopmental disorders.

**Disorder / Context**	**MRI modality**	**Sensitivity**	**Specificity**	**AUC**	**References**
Preschool ASD detection	Quantitative susceptibility, T1 relaxometry	—	—	0.858–0.905	Wang et al., 2026b
Very Preterm, predicting motor outcome	Structural MRI score	78%	78%	—	Mistry et al., 2025
Very Preterm, predicting CP	Term MRI^*^	75%	89%	—	Mistry et al., 2025
Deep learning MRI, ADC for ASD	Combined FLAIR & ADC	85.0%	84.0%	0.898	Guo et al., 2022

^*^Term MRI, refers to MRI acquired at the term equivalent age when imaging preterm infants.

## 6 MRI in guiding intervention and treatment planning

### 6.1 Neuroplasticity insights and monitoring therapy response using MRI biomarkers

MRI increasingly informs intervention strategies for pediatric NDDs by revealing plasticity-sensitive targets. It enables measurement of treatment-induced brain change, guiding rehabilitation planning, and improving prognostic estimates. In children whose brains retain high experience dependent plasticity, MRI can both identify neural systems most amenable to change and provide objective biomarkers. These track the biological impact of therapies, thereby closing the loop between mechanism and clinical practice (Tymofiyeva and Gaschler, 2021). MRI studies of training induced plasticity show that behavioral, educational, and motor therapies produce measurable structural and functional brain changes in young people, and these changes often parallel clinical improvement (Tymofiyeva and Gaschler, 2021). For example, reading, language, and executive function interventions have been associated with increases in task evoked activation in canonical networks, altered resting-state connectivity, and regional structural that correlate with skill gain. Such findings suggest that MRI can identify which circuits are responsive in an individual child and thereby inform the selection and intensity of targeted interventions (Perdue et al., 2022). Longitudinal MRI provides objective endpoints for therapy trials and clinical monitoring. Diffusion metrics (FA, MD, neurite density) capture microstructural remodeling of motor and language tracts following physiotherapy or speech interventions. However, fMRI can quantify normalization or compensation within functional networks after behavioral treatment (Pieri et al., 2021; Yuan et al., 2022). Recent pediatric studies show that DTI changes in corticospinal and association tracts correlate with motor gains in hemiparetic CP, and that task based or resting-state fMRI alterations mirror improvements after targeted cognitive therapies. Radiomic and ML approaches applied to serial MRI add sensitivity by extracting subtle, spatially distributed change patterns that precede or predict behavioral change. These make MRI a valuable biomarker for early detection of responders vs. non-responders in clinical management (Bernstein et al., 2024).

### 6.2 MRI in planning rehabilitation strategies and long-term outcomes

In motor rehabilitation, particularly CP, structural and diffusion MRI inform both prognosis and individualized therapy plans by delineating lesion topography and tract integrity (Yang et al., 2024; Wang Q. et al., 2025). For instance, preserved ipsilesional corticospinal tract microstructure suggests greater potential benefit from constraint induced movement therapy or intensive task practice. Conversely, extensive tract disruption may prompt alternative interventions (orthoses, functional electrical stimulation, or compensatory strategies). Similarly, in language and social communication disorders, MRI indicators such as intact perisylvian white-matter pathways or preserved fronto-temporal functional connectivity can identify children most likely to benefit from intensive language therapy or social skills training. Integrating MRI with clinical assessment thus supports precision rehabilitation planning (Salomon, 2024).

## 7 Challenges and limitations

### 7.1 Practical and ethical considerations for MRI guided intervention

MRI contributes robust prognostic information in several contexts. In neonatal hypoxic ischemic encephalopathy and perinatal brain injury, standardized MRI scoring systems and advanced metrics correlate strongly with later cognitive and motor outcomes. The serial MRI improves predictive accuracy for long-term neurodevelopment. In preterm and congenital risk cohorts, radiomic MRI features and quantitative metrics collected at term equivalent age predict neurodevelopmental impairments at 18 to 36 months (Wu et al., 2023). This is better than demographic or clinical predictors alone. For neurodevelopmental disorders more broadly, combining early MRI signatures with clinical and genetic data yields improved risk models for future functioning, enabling stratified follow up intensity and early allocation of resources (Salomon, 2024). Despite the promise MRI guided personalization faces pragmatic hurdles, the pediatric MRI often requires motion-robust protocols, sedation or natural sleep workflows, and harmonized acquisition across sites to ensure comparable biomarkers. Cost, accessibility, and the risk of acting on uncertain imaging signals must be weighed. The clinicians need validated thresholds that translate imaging change into clinical decision points (Copeland et al., 2021). Ethically, the use of imaging to allocate intensive services raises equity issues unless access is broadly available. Consequently, many authors recommend phased translation. For example, the use MRI as an adjunct in research embedded clinical programs, accumulate longitudinal normative and treatment response datasets, and iteratively refine biomarker thresholds before wide clinical rollout (van der Meulen et al., 2024).

### 7.2 Implementation pathways for MRI

To operationalize MRI in treatment planning, multidisciplinary systems are required such as (a) standardized, pediatric optimized acquisition and preprocessing, (b) normative reference models that account for age and development, (c) validated biomarkers tied to specific intervention responses, and (d) decision support tools that present actionable imaging summaries to clinicians and families (deSouza et al., 2019). Emerging techniques such as quantitative myelin mapping, combined EEG-fMRI, and MRI guided neuromodulation targeting offer avenues to both refine intervention targets and to non-invasively modulate networks identified as dysfunctional. As datasets grow and ML models mature with external validation, MRI will increasingly enable evidence-based, individualized rehabilitation strategies that align neurobiological targets with therapeutic modalities (Wagner et al., 2022). MRI extends beyond its conventional role as a diagnostic imaging in pediatric neurodevelopmental care. When employed longitudinally and integrated with genetic, behavioral, and rehabilitative frameworks, it acts as a dynamic tool to tailor interventions, guide rehabilitation choice, monitor biological response, and refine long-term prognostic predictions. This technique enables the development of truly personalized and mechanism informed therapy in children with NDDs (Parellada et al., 2023). As MRI has great potential for advancing early detection and intervention in pediatric NDDs, its deployment is constrained by several significant challenges. These are ethical concerns associated with imaging children, particularly in research settings (Everts et al., 2022). For instant, the discovery of incidental findings in healthy pediatric volunteers raises complex ethical issues about disclosure, follow-up, and potential anxiety for families. In a study of healthy adolescent volunteers, about 13 % of contributors had incidental lesions, some prompting further clinical workup, and the results demonstrated the tension between research benefit and potential distress (Kumra et al., 2006).

A second major challenge in pediatric MRI is the need for sedation and motion control in young children. MRI is inherently sensitive to motion. However, there are also limitations to acquire to high resolution images in infants and toddlers due to long time period (Barkovich et al., 2019; Copeland et al., 2021). While sedation and general anesthesia can suppress motion, these interventions are associated with potential risks such as neurotoxic effects, airway complications, increased cost, and logistical burdens for recovery (Dong et al., 2019; Badveli et al., 2025). Non-sedative strategies such as scanning infants during natural sleep, using feed and swaddle protocols are increasingly used, but the success rates and institutional adoption is highly variable (Harrington et al., 2022; Greer et al., 2024). Pediatric MRI also faces significant challenges in terms of accessibility, cost, and standardization issues. Protocols usually require specialized tools, longer staff time, and in some cases anesthesia, all of these contribute to higher operational cost. Facilities may incorporate child friendly environment and mock scanners but this infrastructure is not usually available (Harrington et al., 2022; Badveli et al., 2025). Additionally, prolonged scan period pose additional limitation, especially for children with developmental delays, and attempts to decrease protocols can compromise diagnostic quality (Dong et al., 2019; Badveli et al., 2025).

### 7.3 Variability in MRI acquisition and interpretation, and data sharing and integration challenges in research

Variability in MRI acquisition and interpretation in clinical translation poses significant challenges. The differences in scanner models, pulse sequences, coil configuration, and image intensity scales introduce non-biological variables that can obscure or confound true biological signals (Kushol et al., 2023). Additionally, the intensity non-standardness adversely affects the accuracy of image registration and segmentation, making comparisons across subjects and timepoints challenging (Bagci et al., 2010). The clinical interpretation is further hindered by the dynamic nature of pediatric brains and the limited availability standardized normative atlases for very young ages (Barkovich et al., 2019). Finally, data sharing and integration remain significant challenges. Multi-site collaboration is crucial to build sufficiently large pediatric neuroimaging cohorts, but pooling the data faces technical and governance barriers. Variability in data formats, metadata organization, and anonymization practices impedes harmonized sharing. For instance, although platforms such as the Dyslexia Data Consortium have implemented standardized file structures to facilitate data sharing, adoption across the neurodevelopmental field remains inconsistent (Phatangare et al., 2025). The primary challenges limiting the full potential of MRI in NDDs include ethical considerations, high costs, motion control requirements, heterogeneity in acquisition and analysis, and data. Overcoming these limitations will require a concerted effort such as clear ethical frameworks for pediatric imaging, broader implementation of non-sedated imaging protocols, harmonization of image acquisition and processing, standardized, and collaborative data sharing infrastructure.

Although machine-learning approaches applied to pediatric MRI have demonstrated promising performance for early risk stratification of neurodevelopmental disorders, several critical limitations must be acknowledged prior to clinical deployment. The majority of MRI-based classification models are based on statistical effects at the group level, which may not always correspond to accurate diagnostic accuracy at the individual level (Arbabshirani et al., 2017; Poldrack et al., 2020). Furthermore, errors seen in training datasets, such as imbalances in age, sex, ethnicity, socioeconomic position, diagnostic severity, and imaging site, may unintentionally be incorporated by MRI-based models. Because study cohorts in pediatric neuroimaging are frequently selected from financially sound academic institutions and might not accurately reflect the larger clinical population, these biases are especially important (Varoquaux and Cheplygina, 2022).

## 8 Future directions

**Figure 2:**
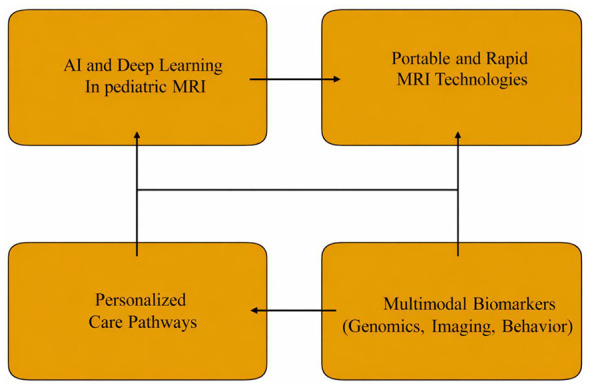
Future directions in MRI driven neurodevelopmental studies.

Looking forward, several emerging trends are expected to significantly advance the application of MRI in early detection and personalized intervention for pediatric NDDs. Particularly, artificial intelligence (AI) and deep learning are anticipated to markedly enhance the sensitivity and scalability of neurodevelopmental MRI. Recent investigation demonstrates that convolutional neural networks, auto encoders, and generative adversarial networks applied to pediatric structural and functional MRI are already achieving high accuracy. Hu and coworkers demonstrated how deep learning architectures are being used to automate feature extraction, enabling end-to-end learning on multimodal pediatric MRI data (Hu et al., 2023). Song and colleagues emphasized that DL models can integrate complex imaging features for early diagnosis of autism and ADHD. It overcomes the limitations of handcrafted feature extraction (Song et al., 2020). Additionally, CNN based models applied to resting-state fMRI in young children have produced near perfect classification of ASD vs. controls, illustrating the power of AI to detect subtle functional connectivity patterns (Feng and Xu, 2023). Development of portable and fast MRI techniques has significant potential for expanding access and greater feasibility in pediatric settings. Developments in hardware and optimized pediatric acquisition strategies are already breaking down barriers to scanning medically fragile children. Another study of pediatric MRI advances demonstrated such innovations as critical for enabling repeated and bedside imaging without heavy sedation (Chen et al., 2025). As these portable systems mature, they may support community based screening, longitudinal monitoring, as well as integration into early intervention programs in underserved regions. Furthermore, there is increasing momentum toward personalized neurodevelopmental care pathways. Rather than using MRI only for diagnosis, future clinical frameworks may integrate imaging derived risk signatures, developmental trajectories, and plasticity potential into tailored therapeutic plans (Mohammad et al., 2025). For instance, AI-derived biomarkers could classify children into subgroups based on their predicted responsiveness to behavioral, pharmacological, and neuromodulatory interventions. This enables precision medicine in a way that aligns neurobiological phenotypes with therapy. Emerging studies of neuroimaging biomarkers in ASD and ADHD suggest that this stratification is within scope, particularly with advances in trans diagnostic and dimensionally informed models (Wen et al., 2024). Future directions in MRI driven for NDDs are shown in Figure 2.

Finally, the convergence of multimodal biomarkers, combining genomics, imaging, and behavioral data, represents a major frontier. Multimodal MRI studies of ASD already integrate diffusion, structural, and perfusion measures, but coupling these with genetic information and longitudinal behavioral profiles could dramatically improve early risk models. Indeed, AI frameworks that fuse imaging with genomic and clinical data are emerging. These enable more precise stratification and potentially revealing mechanistic pathways that underlie individual developmental trajectories. As multimodal databases and collaborative groups grow, such integrative models could be standard tools in early neurodevelopmental screening and personalized care.

## 9 Conclusion

NDDs in children represent a complex and heterogeneous group of conditions arising from disruptions in early brain maturation, with lifelong implications for cognitive, behavioral, and motor functioning. Early detection of NDDs is important as traditional clinical and behavioral assessments often fail to identify subtle neurobiological abnormalities during the critical developmental window. This review demonstrated that MRI has emerged as a transformative tool for understanding the neuropathological underpinnings of NDDs, enabling earlier, more accurate detection and more personalized intervention planning. MRI has great potential to detect structural, functional, and neurochemical deviations from typical developments due to different NDDs such as ASD, ADHD, CP, developmental delay, and genetic or metabolic syndrome. Advance modalities such as diffusion imaging, qMRI, resting-state fMRI, and MRS provide complementary insights into white matter connectivity, cortical organization, brain metabolism, and intrinsic neural network function. These imaging signatures not only enhance the diagnostic precision but also offer potential biomarkers for early risk stratification. Integration of MRI with ML, genomics, and longitudinal clinical data further enhances its predictive power. These multimodal approaches can detect infants and toddlers at high risk for developing NDDs before overt symptoms emerge, potentially revolutionizing early intervention pathways. However, some limitations are also existed particularly, motion artifacts, cost, accessibility, and the lack of standardized acquisition protocols in pediatric settings. Despite these constraints, advancement in AI portable MRI technologies and multimodal biomarker discovery promise to expand the utility of pediatric neuroimaging in the near future.
